# Molecular form and concentration of serum α_2_-macroglobulin in diabetes

**DOI:** 10.1038/s41598-019-49144-7

**Published:** 2019-09-10

**Authors:** Sonomi Yoshino, Kazumi Fujimoto, Tesshu Takada, Sayuki Kawamura, Junro Ogawa, Yuji Kamata, Yoshio Kodera, Masayoshi Shichiri

**Affiliations:** 1 Department of Endocrinology, Diabetes and Metabolism, Kitasato University School of Medicine, 1-15-1 Kitasato, Minami-ku, Sagamihara, Kanagawa 252-0374 Japan; 2 Laboratory of Biomolecular Physics, Department of Physics, & Center for Disease Proteomics, Kitasato University School of Science, 1-15-1 Kitasato, Minami-ku, Sagamihara, Kanagawa 252-0373 Japan; 3 Department of Endocrinology, Diabetes and Metabolism, Kitasato University Medical Center, 6-100 Arai, Kitamoto, Saitama 364-8501 Japan

**Keywords:** Blood proteins, Predictive markers, Cardiovascular diseases, Diabetes complications, Risk factors

## Abstract

α_2_-Macroglobulin is a highly abundant serum protein involved in the development of atherosclerosis and cardiac hypertrophy. However, its circulating molecular form and exact concentrations in human health/diseases are not known. Blue native-polyacrylamide gel electrophoresis of human serum was used to confirm the native conformation of α_2_-macroglobulin. We created an enzyme-linked immunosorbent assay suitable for quantifying its circulating molecular form and undertook a cross-sectional study to measure its serum levels in 248 patients with diabetes mellitus and 59 healthy volunteers. The predominant circulating molecular form of α_2_-macroglobulin was the tetramer, whereas its dimer was detectable in patients with high serum levels of α_2_-macroglobulin. The serum α_2_-macroglobulin concentration was not associated with glycated hemoglobin or any other glycemic variable as evaluated from 48-h continuous glucose monitoring, but showed close correlation with left ventricular posterior wall thickness, carotid artery intima-media thickness, urinary albumin:creatinine ratio (ACR) and brachial–ankle pulse wave velocity (baPWV). Multivariate analysis revealed only the ACR and baPWV to be independent variables influencing serum levels of α_2_-macroglobulin. Thus, an increased ACR and baPWV are associated with higher serum concentrations of α_2_-macroglobulin, and the latter may contribute to the mechanism by which albuminuria increases the risk of developing cardiovascular diseases.

## Introduction

In humans, α_2_-macroglobulin is the largest non-immunoglobulin molecule among the highly abundant proteins in the peripheral blood circulation. α_2_-macroglobulin is synthesized mainly in the liver as a result of coordination between endothelial cells and hepatocytes^1^. α_2_-macroglobulin can inhibit a broad spectrum of serine, threonine, and metalloproteases as well as pro-inflammatory cytokines^2^. It can also induce transcriptional activation of various genes essential for the proliferation/hypertrophy of cells, oncogenesis and atherosclerosis^3^.

Studies using stored pooled human plasma have suggested that two identical α_2_-macroglobulin subunits of size 182 kDa are disulfide-bonded to form dimers, which interact non-covalently to yield a tetrameric structure^4,5^. In biologic fluids, α_2_-macroglobulin tetramers appear to be predominant, but may undergo structural changes during manipulation and preservation^6^. Thus, the exact circulating molecular forms of α_2_-macroglobulin associated with health and diseases have yet to be elucidated.

A 182-kDa protein termed “cardiac isoform of α_2_-macroglobulin” was shown to induce expression of muscle-specific genes associated with the pressure-overloaded heart and to cause cardiac hypertrophy directly^7–10^. This putative “isoform” was claimed to be a key molecule inducing myocardial infarction and cardiac hypertrophy, especially in people with diabetes mellitus (DM) on the basis of serum level measurements^11–13^. Recently, we and others demonstrated using mass spectrometry that the corresponding proteins in rats and humans were indistinguishable from α_2_-macroglobulin molecule^9,14^.

Serum levels of α_2_-macroglobulin were determined first by Ganrot and Scherstén in 1967^15^ and have been shown to be increased in some DM populations, females and certain age groups^15,16^. Increased levels in DM patients were later ascribed mostly to associated conditions, such as microvascular complications^17^, or worsened glycemic control^18–21^. However, those classical studies employed several assay methods using limited numbers of samples, whereas recent studies using commercial enzyme-linked immunosorbent assay (ELISA) kits have reported serum levels of α_2_-macroglobulin to be low^8,11,12,22–25^. The concentrations reported thus far have been very inconsistent, ranging widely over six orders of magnitude^15–23,25–27^. Also, the pathophysiologic role of this factor in human diseases remains unknown.

Microalbuminuria has been measured to assess renal risk in early diabetic nephropathy, but is also a strong predictor of cardiovascular diseases^28–30^. People with type-1 diabetes mellitus (T1DM) and nephropathy carry a tenfold greater risk of adverse cardiovascular outcome compared with those without these disorders^31^. A slightly increased urinary albumin:creatinine ratio (ACR) carries an increased atherosclerotic risk^32^, which is reduced by the regression of albuminuria^28,33^. The latter is also associated with premature death^34–37^ which, in those with T1DM, is due mainly to cardiovascular factors^37^. However, the mechanisms relating albuminuria with an increased cardiovascular risk are not known.

We established a new ELISA to quantify circulating levels of the molecular form of human α_2_-macroglobulin using an antibody that can recognize its monomers, dimers and tetramers in human serum. We designed a cross-sectional study to ascertain if this important molecule is related to diabetic microvascular complications and the risk factors of cardiac/atherosclerotic diseases.

## Results

**Figure 1:**
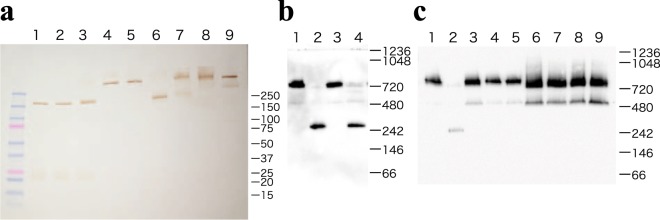
Molecular form of circulating α_2_-macroglobulin molecules in humans. (**a**) Representative SDS-PAGE under reducing and non-reducing conditions of purified human α_2_-macroglobulin protein and human serum. Ten-microliters of fresh human serum diluted to 1:80 and pretreated with 25 mM (lane 1), 10 mM (lane 2), 1 mM (lane 3), 0.1 mM (lane 4), or 0.01 mM of DTT (lane 5), 0.5 µg of purified α_2_-macroglobulin protein pretreated with 25 mM (lane 6) or 0.1 mM of DTT (lane 7), and 10 µL of fresh human serum diluted to 1:80 and pretreated with (lane 8) or without 0.5% SDS (lane 9) were subjected to 4–20% gradient gel electrophoresis and immunoblotted using human α_2_-macroglobulin antibody. (**b**) BN-PAGE of human serum and purified α_2_-macroglobulin protein pretreated with and without DTT. Purified α_2_-macroglobulin protein (0.1 µg) pretreated without (lane 1) or with 25 mM of DTT (lane 2) and 10 μL of fresh human serum diluted to 1:400 pretreated without (lane 3) or with 25 mM of DTT (lane 4) were subjected to BN-PAGE and subsequent immunoblotting using human α_2_-macroglobulin antibody. (**c**) BN-PAGE of purified α_2_-macroglobulin protein and diluted sera and subsequent immunoblotting using human α_2_-macroglobulin antibody. Purified α_2_-macroglobulin (0.05 μg) protein pretreated without (lane 1) or with 25 mM of DTT (lane 2), and 10 µL of diluted sera (1:400) obtained from 3 healthy volunteers (lane 3–5) and 4 patients with diabetes (lane 6–9) showing increased α_2_-macroglobulin concentrations were subjected to BN-PAGE and subsequent immunoblotting. The figure shows the cropped blots and the full-length blots are presented in Supplementary Fig. 1a–c.

To study the native conformation of circulating α_2_-macroglobulin molecules, we incubated purified α_2_-macroglobulin protein and fresh human serum treated with and without dithiothreitol (DTT) and subjected them to sodium dodecyl sulfate (SDS)-PAGE and western blotting. A single band representing α_2_-macroglobulin-like immunoreactivity corresponding to the size of standard α_2_-macroglobulin protein was detected on western blots from healthy human serum. Treatment of serum and an α_2_-macroglobulin standard with increasing concentrations of DTT (1, 10 and 25 mM) reduced the standard α_2_-macroglobulin protein to form monomers of size 182 kDa (Fig. 1a). Blue native (BN)-PAGE of serum and standard α_2_-macroglobulin protein and subsequent immunoblotting revealed a single band corresponding to an α_2_-macroglobulin tetramer of size 725 kDa in untreated samples, whereas treatment of serum and standard α_2_-macroglobulin with DTT reduced the protein into monomers of size 182 kDa (Fig. 1b). A band corresponding to an α_2_-macroglobulin dimer was very faint or barely visible in the lane for a sample of healthy serum or in the lane for standard tetrameric α_2_-macroglobulin. However, in some untreated sera of patients with DM who showed increased serum levels of α_2_-macroglobulin, dimers of α_2_-macroglobulin were visible (Fig. 1c). These results suggested that circulating α_2_-macroglobulin molecules in healthy humans consisted of tetramers that could be monomerized completely by reducing intermolecular disulfide bonds, and that dimers could be present in some patients with DM.

**Figure 2:**
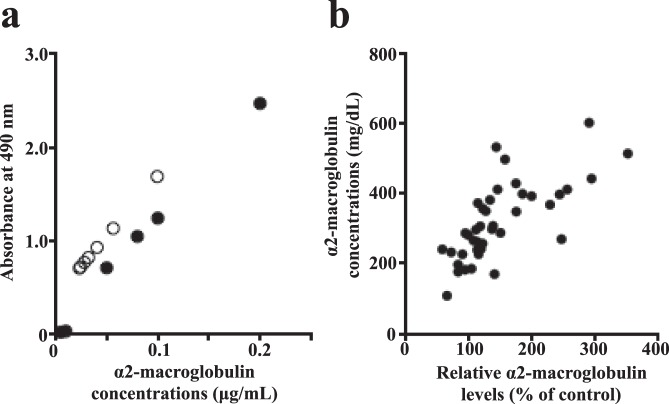
α_2_-macroglobulin ELISA. (**a**) Parallelism of the dilution curve generated by normal human serum serially diluted to 1:40000, 1:80000, 1:120000, 1:160000, 1:200000, 1:240000, and 1:320000 (open circles) with the human α_2_-macroglobulin standard regression curve (closed circles). (**b**) Serum α_2_-macroglobulin levels of 40 DM patients were determined using quantitative western blotting and by ELISA, and the two values were plotted to observe potential correlations.

We established an ELISA using anti-human monoclonal mouse α_2_-macroglobulin IgG, which has been confirmed to recognize monomeric, dimeric and tetrameric α_2_-macroglobulin molecules. A curve prepared with tetrameric α_2_-macroglobulin standards (0.0–0.2 µg/mL) showed parallelism with those of serially diluted serum samples in the ELISA of α_2_-macroglobulin (Fig. 2a). When serial dilutions of serum were applied to the standard curve, a very good correlation was obtained (R^2^ = 0.9646). The concentration of unknown samples was determined by interpolation, which is reliant on a standard curve being generated appropriately. The intra-assay coefficient of variation (CV) of serum samples was 2.3% (n = 8) and inter-assay CV was 13.8% (n = 14). These results suggested that our ELISA was suitable for quantifying serum concentrations of α_2_-macroglobulin in humans. Using serum samples from 40 DM patients, we compared the results obtained with the current ELISA and those determined using quantitative western blotting^14^ (in which α_2_-macroglobulin molecules in serum should undergo reduction/monomerization during SDS-PAGE). Data for the ELISA of α_2_-macroglobulin showed close correlation with the total concentration of monomerized α_2_-macroglobulin (R^2^ = 0.575, p < 0.0001) (Fig. 2b).

**Figure 3:**
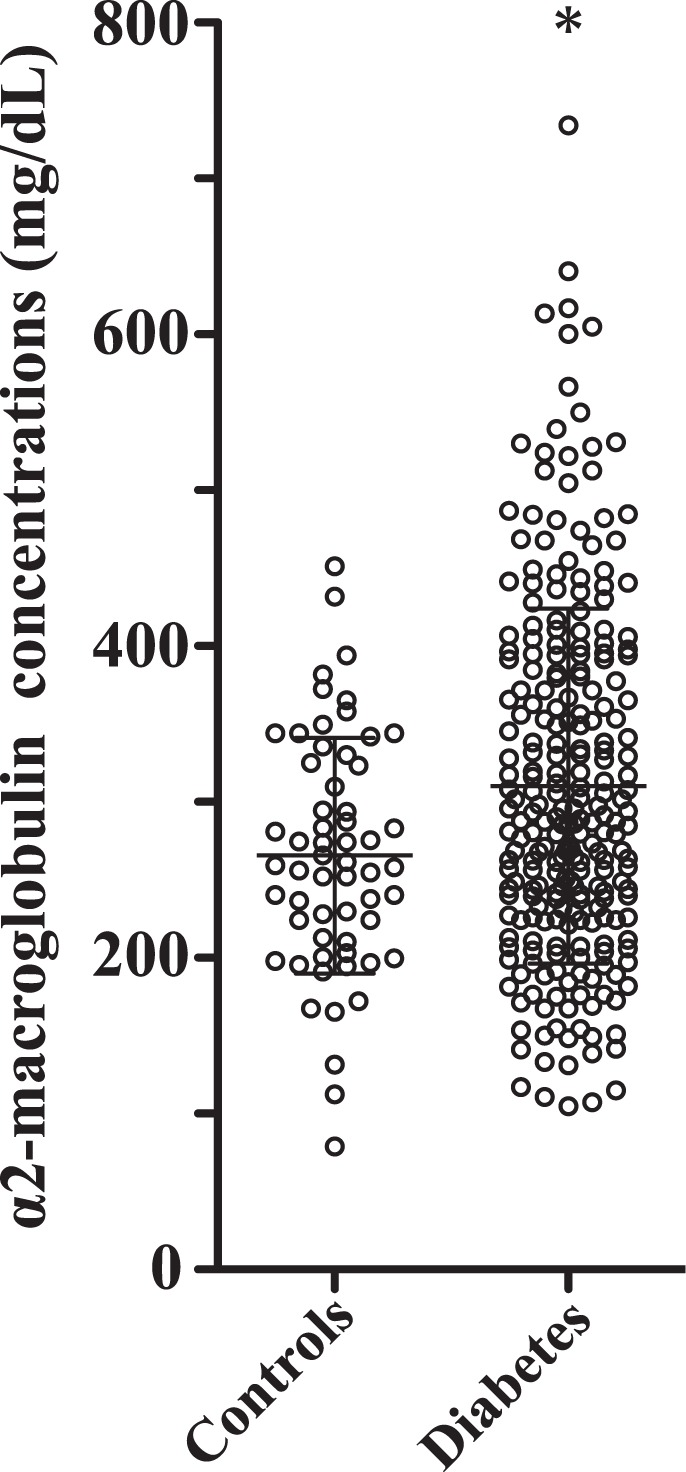
Comparison of serum α_2_-macroglobulin levels in healthy volunteers and in patients with diabetes mellitus. Serum samples obtained from 59 healthy volunteers (Controls) and 248 patients with diabetes were subjected to ELISA of α_2_-macroglobulin. Wide horizontal bars indicate the mean and the shorter horizontal bars indicate the SD. The unpaired *t*-test was used to compare serum levels for the two groups.*p < 0.05 *vs*. controls.

We measured serum concentrations of α_2_-macroglobulin in 59 healthy volunteers and 248 patients with DM (Table 1). Patients with DM showed significantly higher serum levels of α_2_-macroglobulin (310.4 ± 114.2 mg/dL) than healthy volunteers (272.9 ± 76.5 mg/dL) (p = 0.0169) (Fig. 3). When the data of all 307 subjects were analyzed together, serum levels of α_2_-macroglobulin did not correlate with HbA_1c_ level. To confirm no association between serum α_2_-macroglobulin with glycaemic control or glycaemic fluctuation in DM, we analyzed the results of 57 individuals who underwent 48-h CGM at the time of measuring serum levels of α_2_-macroglobulin. Neither the mean glucose level nor data on glycemic fluctuation (standard deviation, CV, mean amplitude of glycemic excursions) showed a correlation with serum levels of α_2_-macroglobulin. These results negate the current belief that serum concentrations of α_2_-macroglobulin reflect glycemic control.

**Table 1:** Characteristics of study participants.

	Healthy volunteers	Diabetes mellitus	p
Number (male/female)	59 (26/33)	248 (133/115)	
Diabetes type (type1/2/gestational)		39/204/5	
Age (years)	54.3 ± 13.2	58.3 ± 15.9	0.0747
Body mass index (kg/m^2^)	22.8 ± 3.4	25.5 ± 5.0	0.0010
HbA_1c_ (%)	5.5 ± 0.3	9.8 ± 2.7	<0.0001
Serum creatinine (mg/dL)	0.71 ± 0.16	0.85 ± 0.42	0.0668
LDL-Cholesterol (mg/dL)	117.3 ± 38.3	115.2 ± 37.0	0.7787
HDL-Cholesterol (mg/dL)	69.1 ± 16.5	51.7 ± 16.0	<0.0001
Triglyceride (mg/dL)	115.1 ± 58.4	162.9 ± 133.5	0.0539
Retinopathy (none/simple/prepro/pro)		180/36/24/8	
Albuminuria categories (^+^1/^+^2/^+^3)		131/74/43	

HDL, high-density lipoprotein; LDL, low-density lipoprotein; HbA_1c_, glycated hemoglobin; ^+^1: albumin:creatinine ratio <30 mg/gCr, ^+^2: 30–299 mg/gCr, ^+^3: >300 mg/gCr.

Pearson’s univariate correlation analysis revealed a positive correlation of serum levels of α_2_-macroglobulin with the ACR, baPWV, maximum intima media thickness (maxIMT), left ventricular posterior wall thickness (LVPWth) and interventricular septum thickness (IVSth), but not with HbA_1c_ levels (Table 2). Serum levels of α_2_-macroglobulin in DM patients with retinopathy were higher than those without (Table 2). Of 248 patients with diabetes, 51 had cardiovascular disease as evidenced by coronary angiography, CT of coronary arteries and/or cardiac ultrasonography. Least square multivariate analyses revealed that only the ACR and baPWV were associated independently with higher serum levels of α_2_-macroglobulin, and that age, sex, eGFR or the presence of retinopathy or cardiovascular disease were not independent variables influencing serum levels of α_2_-macroglobulin (Table 3). Also, serum levels of α_2_-macroglobulin in DM patients with normoalbuminuria (281.0 ± 95.1 mg/dL, n = 131) were not significantly higher than those of healthy volunteers (272.9 ± 76.5 mg/dL, n = 59). Taken together, these results suggested that the ACR and baPWV had an impact on higher serum concentrations of α_2_-macroglobulin.

**Table 2:** Correlation between patient parameters and serum level of α_2_-macroglobulin: univariate analysis.

Parameter	r	P
Age (years)	0.183	0.0013
Male: female		0.0322
BMI (kg/m²)	0.0790	0.1812
HbA_1c_ (%)	−0.0185	0.7616
Retinopathy +/−		0.0020
ACR (mg/gCr)	0.236	0.0002
eGFR (mL/min/1.73 m²)	−0.136	0.0226
baPWV (cm/s)	0.233	0.0005
maxIMT (mm)	0.279	<0.0001
LVPWth (mm)	0.272	0.0039
IVSth (mm)	0.230	0.0139

BMI = body mass index, ACR = albumin:creatinine ratio, eGFR = estimated glomerular filtration rate, baPWV = brachial–ankle pulse wave velocity, maxIMT = maximal intima-media thickness.

LVPWth = left ventricular posterior wall thickness, IVSth = interventricular septum thickness.

**Table 3:** Correlation between characteristics of DM patients and serum level of α_2_-macroglobulin: least square multivariate analysis.

Parameters	β	F	P
Age (years)	0.0929	0.9871	0.3217
Male: female	0.1002	2.0381	0.1550
Retinopathy +/−	−0.0028	0.0014	0.9705
ACR (mg/g)	0.2033	7.4355	0.0070
eGFR (mL/min/1.73 m²)	0.0510	0.3621	0.5480
baPWV (cm/sec)	0.1757	4.1495	0.0430
Cardiovascular disease +/−	0.0864	1.3559	0.2457

ACR = albumin creatinine ratio, eGFR = estimated glomerular filtration rate, baPWV = brachial–ankle pulse wave velocity.

## Discussion

We developed a highly sensitive ELISA to recognize human α_2_-macroglobulin molecules in serum specifically. The limit of detection was 0.5 ng/mL, and the standard curves obtained by diluting the serum from 1:40,000 to 1:320,000 showed parallelism with the standard purified tetramer protein of α_2_-macroglobulin. Inter-assay and intra-assay CVs were small, and the results were stably reproducible. α_2_-macroglobulin levels in human serum have ranged by six orders of magnitude, and reported to be 0.5–2.5 µg/dL when measured using commercial ELISA kits^22,23,25^. Albumin and Ig molecules make up the greatest proportion of plasma proteins, but α_2_-macroglobulin is regarded to be one of top-12 abundant proteins. In the present study, the serum concentration of α_2_-macroglobulin in healthy volunteers was 272.9 ± 76.5 mg/dL, which was quite reasonable.

Considering the well-described potent biologic activities of α_2_-macroglobulin^10,38–41^ and its predominance over other bioactive substances in plasma, significant fluctuations of this important molecule in the peripheral blood circulation could affect the onset and/or progression of human diseases. Our system is the first ELISA suitable for quantifying α_2_-macroglobulin levels in human serum accurately.

BN-PAGE revealed that the α_2_-macroglobulin-like immunoreactivity in human serum migrated to a position corresponding to that of the purified tetramer protein of α_2_-macroglobulin of size 725 kDa. Reduction of the disulfide bonds of purified α_2_-macroglobulin protein and α_2_-macroglobulin molecules in human serum by DTT treatment followed by BN-PAGE and western blotting revealed that α_2_-macroglobulin molecules in serum were separated completely into monomers of size 182 kDa even in the absence of SDS. Furthermore, neither serum nor purified α_2_-macroglobulin protein were monomerized following SDS-PAGE due to non-covalent bonding, whereas both were monomerized completely with reduction of disulfide bonds after DTT treatment. These data argue against the current belief based on studies using stored human plasma that two disulfide-bonded α_2_-macroglobulin dimers are non-covalently bonded to form tetramers in the peripheral blood circulation^2,4,5,42^.

In some patients with DM whose serum α_2_-macroglobulin levels were increased markedly, dimeric components were observed in addition to tetramers according to BN-PAGE. Thus, dimers of α_2_-macroglobulin may be formed in human blood if α_2_-macroglobulin synthesis is enhanced. Our results demonstrate that the α_2_-macroglobulin antibody that we used had cross-reactivity with monomers, dimers and tetramers, which could be present in human serum. We compared the results of semiquantitative measurement of serum α_2_-macroglobulin monomerized after DTT treatment and SDS-PAGE with measurements by ELISA using 40 samples of human serum, and found that both were highly correlated (Fig. 2b). We concluded that our ELISA reflected the circulating components of human α_2_-macroglobulin molecules.

We also found significantly high serum levels of α_2_-macroglobulin in DM patients compared with healthy volunteers. Several studies have reported a positive correlation between the HbA_1c_ level and α_2_-macroglobulin level in serum^20–22^, but we did not. To negate the possibility that blood glucose levels in DM affect serum concentrations of α_2_-macroglobulin, we obtained the exact glycemic profiles of 57 individuals through 48-h CGM. Neither the mean blood glucose levels of the entire 48-h recordings nor variables of glycemic fluctuation (standard deviation, CV) correlated with serum levels of α_2_-macroglobulin. Univariate regression analysis of 248 DM patients revealed that the α_2_-macroglobulin level correlated with the ACR, baPWV, maxIMT, LVPWth, IVSth, but not with the HbA_1c_ level. Serum levels of α_2_-macroglobulin were significantly higher in DM patients with retinopathy than those without retinopathy. Among these variables, however, multiple regression analysis with the α_2_-macroglobulin level as the objective variable revealed only the ACR and baPWV to be significant independent variables affecting the serum level of α_2_-macroglobulin. The association of the urinary albumin concentration with an increased level of this important molecule may be linked to the mechanism by which albuminuria increases the risk of developing cardiac and atherosclerotic diseases.

Albuminuria was originally identified as an incipient marker of diabetic nephropathy^43,44^, but is now well documented as an independent predictor of cardiovascular morbidity and mortality not only in T1DM and T2DM^28,37,45,46^, but across varied non-DM populations^29,36,47,48^. Accumulating evidence suggests that proteinuria is a stronger predictor of cardiovascular disease than traditional risk factors such as cholesterol and blood pressure^30,49^, whereas albuminuria is associated with an increased risk starting well below the cutoff for microalbuminuria^29,48,49^. However, the exact pathophysiologic conditions responsible for the overwhelming cardiovascular consequences induced specifically by trace albuminuria, but not in patients without albuminuria, have remained unknown to date.

**Figure 4:**
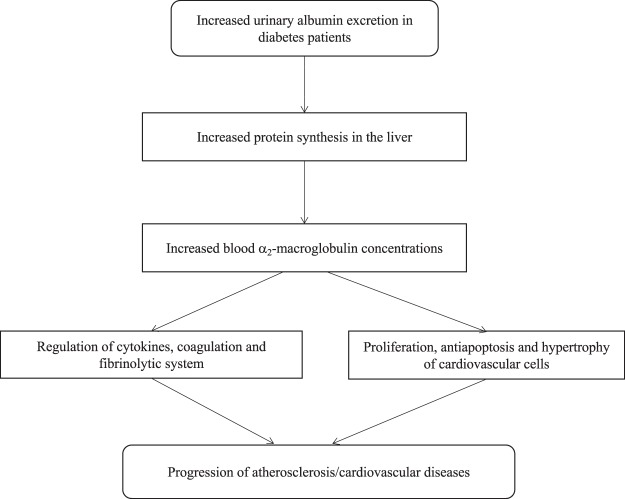
Flowchart for the proposed mechanistic link between albuminuria, serum α_2_-macroglobulin and cardiovascular disease in diabetes.

Several mechanisms have been proposed to contribute to the cardiovascular consequences of albuminuria, including inflammation, endothelial dysfunction, thrombogenic factors, and insulin resistance^50–53^. In nephrotic syndrome, hepatic synthesis of the high-molecular-weight protein α_2_-macroglobulin has been shown to be enhanced significantly to replace lost liver-derived proteins in experimental animals^54^ and humans^55^, resulting in a net increase in its serum levels^54^. The present study demonstrated a close correlation between serum levels of α_2_-macroglobulin and the ACR, and that the former start to rise when a trace amount of albumin is excreted. Considering the magnitude of increased post-transcriptional α_2_-macroglobulin synthesis in the liver in response to urinary protein excretion^54–56^, it is reasonable to assume that circulating α_2_-macroglobulin levels may be increased far more profoundly by urinary albumin than by other factors such as sex, age, glycemic control or diabetic retinopathy. A proposed mechanistic link relating albuminuria to increased serum level of α_2_-macroglobulin and cardiovascular disease in diabetes is depicted in Fig. 4.

Considering the limited number of individuals employed in this cross-sectional study, whether an increased serum level of α_2_-macroglobulin in diabetes mellitus could serve as a mediator of albuminuria that, ultimately, results in cardiovascular diseases, is not known. Also, whether preventive therapy can reverse albuminuria and reduce the serum level of α_2_-macroglobulin is not known. Further studies to ascertain if measurement of the serum level of α_2_-macroglobulin could help to identify patients at a high risk of cardiovascular disease should be carried out.

In conclusion, using a sensitive and specific ELISA, we identified, among other laboratory parameters, a close association between serum levels of α_2_-macroglobulin with ACR and baPWV. Measurement of serum levels of α_2_-macroglobulin could help to identify patients at high risk of cardiovascular diseases.

## Methods

### Study participants

We enrolled 248 patients with DM (39 with T1DM, 204 with T2DM, and 5 with gestational DM) and 59 healthy volunteers at Kitasato University Medical Center or Kitasato University Hospital. Excluded from the analyses were patients with infectious diseases, malignancy, end-stage renal failure, autoimmune thyroid diseases and those receiving corticosteroids. We obtained information on the medical and life histories of all participants.

Patients underwent routine evaluation of systemic diseases covered by the universal health coverage system in Japan^57,58^. These included electrocardiography (ECG), radiography (chest and abdomen), ultrasonography (neck and/or abdomen), urinalysis, complete blood count, biochemical analyses of serum, as well as tests for levels of free thyroxine, free triiodothyronine, thyroid-stimulating hormone (TSH) and thyroid antibodies. DM patients underwent ophthalmologic and neurologic tests, the anti-glutamic acid decarboxylase antibody test as well as measurements of glycated albumin and/or glycated hemoglobin (HbA_1c_), fasting serum insulin, and urinary ACR.

Glycemic control/fluctuation was assessed in 54 DM patients without macroalbuminuria (18 males and 36 females; 41.3 ± 13.5 years; HbA_1c_, 8.0 ± 1.8%) and 3 people without DM (3 females; 40.0 ± 2.6 years; 5.7 ± 0.2%) using 48-h continuous glucose monitoring (CGM) recording (CGMS^®^ GOLD, Medtronic Minimed, Northridge, CA, USA)^59,60^.

Cardiovascular diseases were diagnosed by ECG, exercise electrocardiography, ankle–brachial pressure index, brachial–ankle pulse wave velocity (baPWV), cardiac catheterization/angiography, and/or coronary computed tomography. For healthy volunteers, the blood-test data at the most recent medical checkup were used to confirm the absence of other systemic diseases and as analytical data.

### Collection of serum samples

Blood samples from study participants were collected into vacutainers. Serum was separated immediately in a refrigerated centrifuge and stored in aliquots at −30 °C until processing.

### Materials

Purified human α_2_-macroglobulin protein (725 kDa) was obtained from Enzo Life Science (New York, USA), anti-human monoclonal mouse α_2_-macroglobulin (immunoglobulin (Ig)G1 clone) from R&D Systems (Minneapolis, MN, USA), and goat anti-mouse IgG (H + L)-horseradish peroxidase (HRP) conjugate from Bio-Rad Laboratories (Hercules, CA, USA). Native PAGE™ Sample Buffer, Native PAGE Running Buffer, Dark Blue Cathode Buffer, Native Mark^™^ Unstained Protein Standard, and Light Blue Cathode Buffer were obtained from Thermo Fisher Scientific (Waltham, MA, USA). DTT, Blocking One and Peroxidase Stain 3,3′-Diaminobenzidine kit (Brown Stain) were purchased from Nacalai Tesque (Kyoto, Japan). Immune-Blot^®^ polyvinylidene difluoride membrane, Sequi-Blot™ membrane, Precision Plus Protein™ Dual Color Standards and Mini-PROTEAN^®^ TGX™ were obtained from Bio-Rad Laboratories. Perfect NT Gel, Perfect NT Gel System and SDS-PAGE Running Buffer were obtained from DRC (Tokyo, Japan), and ECL Prime Western Blotting Detection Reagent from GE Healthcare (Buckinghamshire, UK).

### ELISA of α_2_-macroglobulin

All measurements were carried out in triplicate. One-hundred microliters of human α_2_-macroglobulin protein diluted to 0.5–80 ng/mL and serum samples diluted to 1:160,000 with phosphate-buffered saline (PBS) were incubated overnight at 4 °C in a 96-well microplate. Plates were washed thrice and blocked with 200 µL/well of PBS containing 0.05% Tween^®^20 (PBS-T) and 5% (*w/v*) skimmed milk/Tris-buffered saline (TBS) for 2 h at room temperature. After washing thrice with PBS-T (200 µL/well), plates were incubated overnight with 100 µL of anti-human monoclonal mouse α_2_-macroglobulin at 1:5,000 dilution. Then, plates were washed thrice with PBS-T (200 µL/well) and reacted with 1:10,000 goat anti-mouse IgG (H + L)-HRP conjugate (100 µL/well) and allowed to stand at room temperature for 1 h under light-shielding. After washing four times with PBS-T (200 µL/well), *o*-phenylenediamine/0.05 M phosphate-citrate buffer (100 µL/well) was added for 30 min at room temperature under light-shielding, then overlaid with 3 N HCl (100 µL/well) to terminate the reaction. Absorbance of the plate was measured at 490 nm using a microplate reader (iMark™; Bio-Rad Laboratories).

### Polyacrylamide gel electrophoresis (PAGE) followed by western blotting

SDS-PAGE was performed using purified human α_2_-macroglobulin protein and human serum essentially as described^61,62^ except for the following modifications. Ten-microliters of fresh human serum diluted to 1:80 and 0.5 µg of purified α_2_-macroglobulin protein pretreated with or without DTT were subjected to 4–20% gradient gel electrophoresis using Mini-PROTEAN^®^ TGX™ and subsequent immunoblotting using human α_2_-macroglobulin antibody.

Blue native PAGE was performed as described^63^ except for the following modifications. Native PAGE™ Sample Buffer was used to dissolve 0.1 µg of standard α_2_-macroglobulin and to dilute 0.025 µL of serum. After pretreatment with or without 25 mM of DTT, 10 µL of diluted samples was loaded on a 5–20% gradient Perfect NT Gel using Native PAGE Running Buffer as a positive electrode and Dark Blue Cathode Buffer as a negative electrode according to manufacturer instructions. Native Mark^™^ Unstained Protein Standard was used as a marker of molecular size. After migrating samples at 150 V to one-third of the entire gel, Dark Blue Cathode Buffer in the negative electrode was replaced with Light Blue Cathode Buffer to complete the remaining electrophoresis.

After electrophoresis, the gel was photographed, transferred to a PVDF membrane (Immune-Blot^®^) at 75 V for 120 min, incubated with 8% acetic acid for 15 min, then with sterilized water for 5 min, and air-dried to fix the protein. The PVDF membrane was then immersed in MeOH to remove Coomassie brilliant blue G-250 dye. Then, it was washed with sterilized water, blocked at 4 °C overnight with Blocking One and, after washing thrice with TBS-T, incubated overnight with human α_2_-macroglobulin antibody (1:5000 dilution) at 4 °C. After incubation with goat anti-mouse IgG (H + L)-HRP Conjugate (1:10000 dilution) for 1 h, protein bands were detected using ECL and photographed and analyzed with ImageQuant LAS 4000 (GE Healthcare).

### Quantitative western blotting to measure levels of monomerized α_2_-macroglobulin

Serum samples from 40 consecutive DM patients were diluted 40-fold with binding buffer (50 mM DTT, 0.5% SDS, 10% glycerol/1 M Tris-HCl) and subjected to SDS-PAGE with a protein marker (Precision Plus Protein™ Dual Color Standards) using a 5% Perfect NT Gel and a Perfect NT Gel System SDS-PAGE Running Buffer at 120 V for 60–90 min. Proteins in the gels were then transferred to a PVDF membrane (Sequi-Blot™) at 60 mA for 60 min and incubated overnight at 4 °C with Blocking One. After washing thrice with TBS-T, the PVDF membrane was incubated with human α_2_-macroglobulin antibody (1:3000 dilution) for 90 min, and then with goat anti-mouse IgG(H + L)-HRP Conjugate (1:5000 dilution) for 1 h at room temperature. The PVDF membrane was visualized using Brown Stain. The intensity of each band was quantified using the ImageQuant TL program (GE Healthcare). All densitometric data were converted to relative monomer concentrations using twofold serially diluted samples of control protein.

### Statistical analyses

Data are the mean ± SD unless indicated otherwise. Statistical analyses were carried out using JMP v5 (SAS institute, Cary, NC, USA) and Prism 5 (GraphPad Software, La Jolla, CA, USA). Differences between groups were examined for significance using the unpaired *t*-test. Correlation analysis was undertaken on the relationship between the α_2_-macroglobulin level and each parameter. Multiple regression analyses were done employing age, sex, diabetic retinopathy, the ACR, estimated glomerular filtration rate (eGFR), baPWV and cardiovascular disease as an explanatory variable and serum level of α_2_-macroglobulin as an objective variable. *P* < 0.05 was considered significant.

### Ethics approval and consent to participate

The study protocol was approved by the Ethics Committees of Kitasato University Medical Center (Saitama, Japan; 29–27) and Kitasato University Hospital (Kanagawa, Japan; B17-119). All study methods were undertaken in accordance with the relevant guidelines and regulations of these two organizations as well as the *Ethical Guidelines for Medical and Health Research Involving Human Subjects in Japan*. Written informed consent was obtained from all participants.

## Data Availability

All data generated or analyzed during this study are included in this article.

## References

[1] Talamini MA, McCluskey MP, Buchman TG, De Maio A (1998). Expression of alpha2-macroglobulin by the interaction between hepatocytes and endothelial cells in coculture. Am J Physiol.

[2] Rehman AA, Ahsan H, Khan FH (2013). Alpha-2-macroglobulin: a physiological guardian. J Cell Physiol.

[3] Gopal U, Gonzalez-Gronow M, Pizzo SV (2016). Activated alpha2-macroglobulin regulates transcriptional activation of c-myc target genes through cell surface GRP78 protein. J Biol Chem.

[4] Jensen PE, Sottrup-Jensen L (1986). Primary structure of human alpha 2-macroglobulin. Complete disulfide bridge assignment and localization of two interchain bridges in the dimeric proteinase binding unit. J Biol Chem.

[5] Sottrup-Jensen L (1983). The primary structure of alpha 2-macroglobulin and localization of a Factor XIIIa cross-linking site. Ann N Y Acad Sci.

[6] Wyatt AR, Kumita JR, Farrawell NE, Dobson CM, Wilson MR (2015). Alpha-2-macroglobulin is acutely sensitive to freezing and lyophilization: Implications for structural and functional studies. PLoS One.

[7] Mariappan M, Selvamurugan N, Rajamanickam C (1990). Purification and characterization of a high-molecular-weight protein induced in rat serum during the development of cardiac hypertrophy. Arch Biochem Biophys.

[8] Prabhakar R, Rajamanickam C (1993). Serum protein of 135-kDa molecular weight-a molecular signal for cardiac hypertrophy. Arch Biochem Biophys.

[9] Rajamanickam C, Sakthivel S, Babu GJ, Lottspeich F, Kadenbach B (2001). Cardiac isoform of alpha-2 macroglobin, a novel serum protein, may induce cardiac hypertrophy in rats. Basic Res Cardiol.

[10] Rajan S, Radhakrishnan J, Rajamanickam C (2003). Direct injection and expression *in vivo* of full-length cDNA of the cardiac isoform of alpha-2 macroglobulin induces cardiac hypertrophy in the rat heart. Basic Res Cardiol.

[11] Annapoorani P (2006). Cardiac isoform of alpha-2 macroglobulin-a new biomarker for myocardial infarcted diabetic patients. Atherosclerosis.

[12] Rajamanickam C, Sakthivel S, Kurian Joseph P, Athimoolam Janarthanan R (1998). A novel serum protein of molecular weight 182 kDa: a molecular marker for an early detection of increased left ventricular mass in patients with cardiac hypertrophy. J Cardiovasc Risk.

[13] Soman S, Manju CS, Rauf AA, Indira M, Rajamanickam C (2011). Role of cardiac isoform of alpha-2 macroglobulin in diabetic myocardium. Mol Cell Biochem.

[14] Takada T (2013). Serum monomeric alpha2-macroglobulin as a clinical biomarker in diabetes. Atherosclerosis.

[15] Ganrot PO, Scherstén B (1967). Serum α2-macroglobulin concentration and its variation with age and sex. Clin Chim Acta.

[16] Tunstall AM, Merriman JM, Milne I, James K (1975). Normal and pathological serum levels of alpha2-macroglobulins in men and mice. J Clin Pathol.

[17] Ahmad J, Singh M, Saleemuddin M (2001). A study of plasma alpha-2-macroglobulin levels in type 2 diabetic subjects with microalbuminuria. J Assoc Physicians India.

[18] Ceriello A (1989). Increased alpha 2-macroglobulin in diabetes: a hyperglycemia related phenomenon associated with reduced antithrombin III activity. Acta Diabetol Lat.

[19] Gray RS (1982). Alpha 2-macroglobulin and proliferative retinopathy in type 1 diabetes. Horm Metab Res.

[20] James K, Merriman J, Gray RS, Duncan LJ, Herd R (1980). Serum alpha 2-macroglobulin levels in diabetes. J Clin Pathol.

[21] Lisowska-Myjak B, Pachecka J, Kaczynska B, Miszkurka G, Kadziela K (2006). Serum protease inhibitor concentrations and total antitrypsin activity in diabetic and non-diabetic children during adolescence. Acta Diabetol.

[22] Chung TJ (2016). Association of salivary alpha 2-macroglobulin levels and clinical characteristics in type 2 diabetes. J Diabetes Investig.

[23] Dursun E (2015). The interleukin 1 alpha, interleukin 1 beta, interleukin 6 and alpha-2-macroglobulin serum levels in patients with early or late onset Alzheimer’s disease, mild cognitive impairment or Parkinson’s disease. J Neuroimmunol.

[24] Meenakshisundaram R, Sweni S, Thirumalaikolundusubramanian P (2010). Cardiac isoform of alpha 2 macroglobulin: a marker of cardiac involvement in pediatric HIV and AIDS. Pediatr Cardiol.

[25] Zabel M (2012). Assessing candidate serum biomarkers for Alzheimer’s disease: a longitudinal study. J Alzheimers Dis.

[26] Thieme R (2015). Analysis of alpha-2 macroglobulin from the long-lived and cancer-resistant naked mole-rat and human plasma. PLoS One.

[27] Varma VR (2017). Alpha-2 macroglobulin in Alzheimer’s disease: a marker of neuronal injury through the RCAN1 pathway. Mol Psychiatry.

[28] de Zeeuw D (2004). Albuminuria, a therapeutic target for cardiovascular protection in type 2 diabetic patients with nephropathy. Circulation.

[29] Gerstein HC (2001). Albuminuria and risk of cardiovascular events, death, and heart failure in diabetic and nondiabetic individuals. JAMA.

[30] Ljungman S, Wikstrand J, Hartford M, Berglund G (1996). Urinary albumin excretion-a predictor of risk of cardiovascular disease. A prospective 10-year follow-up of middle-aged nondiabetic normal and hypertensive men. Am J Hypertens.

[31] Tuomilehto J (1998). Incidence of cardiovascular disease in Type 1 (insulin-dependent) diabetic subjects with and without diabetic nephropathy in Finland. Diabetologia.

[32] Deckert T (1996). Cohort study of predictive value of urinary albumin excretion for atherosclerotic vascular disease in patients with insulin dependent diabetes. BMJ.

[33] Jansson FJ (2018). Regression of albuminuria and its association with incident cardiovascular outcomes and mortality in type 1 diabetes: the FinnDiane Study. Diabetologia.

[34] Borch-Johnsen K, Kreiner S (1987). Proteinuria: value as predictor of cardiovascular mortality in insulin dependent diabetes mellitus. BMJ.

[35] Groop PH (2009). The presence and severity of chronic kidney disease predicts all-cause mortality in type 1 diabetes. Diabetes.

[36] Hillege HL (2002). Urinary albumin excretion predicts cardiovascular and noncardiovascular mortality in general population. Circulation.

[37] Rossing P, Hougaard P, Borch-Johnsen K, Parving HH (1996). Predictors of mortality in insulin dependent diabetes: 10 year observational follow up study. BMJ.

[38] Brownlee M (1976). α_2_- macroglobulin and reduced basement -membrane degradation inn diabetes. Lancet.

[39] Hubbard WJ (1978). Alpha-2 macroglobulin-enzyme complexes as suppressors of cellular activity. Cell Immunol.

[40] Koo PH (1983). Human alpha 2-macroglobulin: a major serum factor cytotoxic for tumor cells. Cancer Lett.

[41] Tiggelman AM, Linthorst C, Boers W, Brand HS, Chamuleau RA (1997). Transforming growth factor-beta-induced collagen synthesis by human liver myofibroblasts is inhibited by alpha2-macroglobulin. J Hepatol.

[42] Marrero A (2012). The crystal structure of human alpha2-macroglobulin reveals a unique molecular cage. Angew Chem Int Ed Engl.

[43] Mogensen CE (1984). Microalbuminuria predicts clinical proteinuria and early mortality in maturity-onset diabetes. N Engl J Med.

[44] Viberti GC (1982). Microalbuminuria as a predictor of clinical nephropathy in insulin-dependent diabetes mellitus. Lancet.

[45] Dinneen SF, Gerstein HC (1997). The association of microalbuminuria and mortality in non-insulin-dependent diabetes mellitus. A systematic overview of the literature. Arch Intern Med.

[46] Morrish NJ, Stevens LK, Fuller JH, Jarrett RJ, Keen H (1991). Risk factors for macrovascular disease in diabetes mellitus: the London follow-up to the WHO Multinational Study of Vascular Disease in Diabetics. Diabetologia.

[47] Damsgaard EM, Froland A, Jorgensen OD, Mogensen CE (1990). Microalbuminuria as predictor of increased mortality in elderly people. BMJ.

[48] Wachtell K (2003). Albuminuria and cardiovascular risk in hypertensive patients with left ventricular hypertrophy: the LIFE study. Ann Intern Med.

[49] Perkovic V (2008). The relationship between proteinuria and coronary risk: a systematic review and meta-analysis. PLoS Med.

[50] Deckert T, Feldt-Rasmussen B, Borch-Johnsen K, Jensen T, Kofoed-Enevoldsen A (1989). Albuminuria reflects widespread vascular damage. The Steno hypothesis. Diabetologia.

[51] Mykkanen L (1998). Microalbuminuria is associated with insulin resistance in nondiabetic subjects: the insulin resistance atherosclerosis study. Diabetes.

[52] Pedrinelli R (1994). Microalbuminuria and endothelial dysfunction in essential hypertension. Lancet.

[53] Stehouwer CD (2002). Increased urinary albumin excretion, endothelial dysfunction, and chronic low-grade inflammation in type 2 diabetes: progressive, interrelated, and independently associated with risk of death. Diabetes.

[54] Stevenson FT, Greene S, Kaysen GA (1998). Serum alpha 2-macroglobulin and alpha 1-inhibitor 3 concentrations are increased in hypoalbuminemia by post-transcriptional mechanisms. Kidney Int.

[55] de Sain-van der Velden MG (1998). Plasma alpha 2 macroglobulin is increased in nephrotic patients as a result of increased synthesis alone. Kidney Int.

[56] de Sain-van der Velden MG (2000). Nephrotic proteinuria has No net effect on total body protein synthesis: measurements with (13)C valine. Am J Kidney Dis.

[57] Chida S (2016). Levels of albuminuria and risk of developing macroalbuminuria in type 2 diabetes: historical cohort study. Sci Rep.

[58] Kamata Y (2017). Distinct clinical characteristics and therapeutic modalities for diabetic ketoacidosis in type 1 and type 2 diabetes mellitus. J Diabetes Compl.

[59] Ogawa A (2012). New indices for predicting glycaemic variability. PLoS One.

[60] Hayashi A (2016). Distinct biomarker roles for HbA1c and glycated albumin in patients with type 2 diabetes on hemodialysis. J Diabetes Compl.

[61] Shichiri M, Nonaka D, Lee LJ, Tanaka K (2018). Identification of the salusin-beta receptor using proteoliposomes embedded with endogenous membrane proteins. Sci Rep.

[62] Tani Y, Yamada S, Inoshita N, Hirata Y, Shichiri M (2015). Regulation of growth hormone secretion by (pro)renin receptor. Sci Rep.

[63] Hoshiyama, A. *et al*. Identification of plasma binding proteins for glucose-dependent insulinotropic polypeptide. *Endocrine J*, in press (2019). 10.1507/endocrj.EJ18-0472 31061246

